# TRS-PCR profiling for discrimination of *Escherichia coli* strains isolated from children with diarrhea under 5 years of age in Lodz region, Poland

**DOI:** 10.1007/s11033-016-4031-x

**Published:** 2016-07-07

**Authors:** Anna B. Kubiak-Szeligowska, Milena Bartnicka, Dariusz Jarych, Marta Majchrzak

**Affiliations:** 1 Institute of Medical Biology, Polish Academy of Sciences, 106 Lodowa, Str., 93-232 Lodz, Poland; 2 Laboratory of Personalized Medicine, Lodz Regional Science and Technology Park Ltd., 114/116 Dubois Str., 93-465 Lodz, Poland

**Keywords:** Children’s diarrhea, *Escherichia coli*, TRS-PCR, Genotyping

## Abstract

*Escherichia coli* is one of the most frequently isolated gram-negative pathogens in cases of foodborne diseases and hospital infections. What is more, diarrheal diseases, including these associated with pathogenic *E. coli* strains, are leading causes of morbidity and mortality worldwide, especially among children. Improvements of the management of diarrheal diseases caused by these bacteria are in the spotlight of the World Health Organization. Therefore, there is still a need to develop new methods or improve ones that are commonly used to characterize and distinguish *E. coli* strains more precisely. In this work, TRS-based PCRs were effectively used for discrimination of 123 *E. coli* strains isolated from children with diarrhea in the Lodz region (Poland). The composite TRS-PCR approach, based on similarity comparisons of GTG-PCR and CGG-PCR fingerprints, enabled us to distinguish strains with very good efficacy. This was confirmed by the high diversity index (0.991) and high reproducibility of the band patterns obtained (95.0 %). These results showed the great variation in strains that may cause infections in children under 38 months. However, the stains were grouped in three separate clusters, which were different in terms of their phylogenetic affiliation and virulence factor repertoire. The obtained results support and are consistent with the need of public health surveillance for searching new and fast assays as far as children’s health is concerned. TRS-PCR profiling is an effective tool for genotyping of *E. coli* strains isolated from children with diarrhea.

## Introduction

Diarrheal diseases are common not only in developing countries but also in industrialized ones, resulting in morbidity and mortality globally, especially among children [1, 2]. According to the World Health Organization (WHO), diarrhea and pneumonia kill 2 million children under 5 years of age every year, of which diarrhea is the cause of up to 11 % of the deaths [3]. In 2013, 6.3 million children died before their fifth birthday and 9.2 % of the deaths were caused by diarrhea (0.578 million cases) [4]. The etiology of the diarrhea differs in terms of region or population [2], and often its cause remains unidentified [1]. In the case of human enteric infections, *Escherichia coli* is an important and widespread pathogen [1, 5–7].

*Escherichia coli* is a highly diverse bacterial species, including intestinal and extraintestinal pathogens. Both groups are further subdivided into pathotypes. A given pathotype can be stated based on many features, such as the presence of specific virulence factors, inducement of specific clinical symptoms, O-serotype and phylogenetic grouping [1, 6–8]. Diarrheagenic *E. coli* (DEC) strains are generally subdivided into seven or eight major groups [1, 5, 7]. Among them, enteropathogenic (EPEC), enterotoxigenic (ETEC) and enteroaggregative (EAEC) *E. coli* are major causes of children’s diarrhea [1, 5]. Such infections often have fatal consequences in developing countries but are mild and self-limiting in industrialized countries [1]. Atypical enteropathogenic *E. coli* (aEPEC) strains seem to have a predisposition to cause persistent diarrhea [1] and are more frequently isolated in the developed world than EPEC [5, 7]. Infections due to Shiga Toxin producing *E. coli* (STEC) (including enterohemorrhagic *E. coli* strains, EHEC) are relatively rare but a wide spectrum of illnesses and high mortality rates make these bacteria emerging pathogens [1, 5]. Some researchers emphasize that the role of DEC strains as far as sporadic pediatric diarrhea is concerned is still under-recognized in developed countries [2]. Moreover, it is known that sometimes a detected enteric pathogen in a child with diarrhea might not be the cause of the illness [9]. Two of five of WHO’s main goals presented in The Integrated Global Action Plan for the Prevention and Control of Pneumonia and Diarrhoea (GAPPD) are concentrated on reducing mortality from diarrhea and reducing the incidence of severe diarrhea in children under 5 years of age by 2025 [3]. It may be easier to achieve these goals by conducting studies based on better characterization of DECs, which further may be useful in implementation of improvements in public health monitoring under non-epidemic conditions [2].

Nowadays, detection of DECs and their distinction from commensal *E. coli* is based on combination of biochemical tests, serotyping, virulence profiling and various molecular methods [2, 5]. Huge progress has been made in detection of enteropathogens, but molecular biology techniques, even PCR-based assays are generally limited to reference laboratories and specifically for outbreak investigations [2]. As there is still a need for simple, fast and inexpensive methods for discrimination of DEC strains when there is no ongoing outbreak [2], we present the usefulness of the TRS-PCR-based technique in distinguishing *E. coli* strains isolated from children with diarrhea in the Lodz region of Poland.

## Materials and methods

### Bacterial strains

All strains were isolated from children with diarrhea in the Lodz region (Poland) and were obtained from the Medical Laboratory SYNEVO in Lodz, Poland. Isolates were collected from January 2009 to May 2010. In total, 123 of *E. coli* strains originated from stool samples. Pure cultures were characterized biochemically and resistance to antibiotics was specified (SYNEVO). The strains used in this study were also examined genetically and were serotyped according to manufacturer’s protocols for *E. coli* (O pool and O single antisera, Statens Serum Institut SSI Diagnostica, Denmark). Detailed characteristics of the collection of strains is presented in Table 1.

**Table 1:** *E. coli* isolates used in this study

Cluster	Key	Co-infection	Patient’s sex^a^	Patient’s age (months)	Place of isolation^b^	Serotype^c^	Phylogenetic group	Virulence profile
I	K 037	–	M	13	4	O25	A	(−)
	K 123	–	M	1	3	O25	A	(−)
	K 135	*E. coli* O125, fungi	F	11	4	O125	A	*fimG/fimH*
	K 098	–	M	13	2	O128	A	*fimG/fimH*
	K 103	*E. coli* O128, fungi	F	21	2	O142	B1	*fimG/fimH*
	K 118	Fungi	M	30	4	O128	B1	*fimG/fimH*
	K 057	–	F	9	4	O25	B1	*fimG/fimH*
	K 053	Fungi	M	31	4	O86	B1	*fimG/fimH*
	K 162	–	M	34	4	O55	A	*fimG/fimH*
	K 048	–	M	20	3	O128	A	*fimG/fimH*
	K 100	Fungi	M	9	4	O128	A	*fimG/fimH*
	K 007	MSSA	M	7	4	O126	A	*fimG/fimH*
	K 121	Fungi	M	23	3	O111	B1	*fimG/fimH, escV*
	K 133	MRSA, fungi	F	17	4	O128	B1	*fimG/fimH, escV*
	K 032	Fungi	M	1	4	O128	B1	*fimG/fimH, escV*
	K 160	–	F	12	4	O128	B1	*fimG/fimH, escV*
	K 128	–	F	36	4	O55	E	*fimG/fimH, escV*
	K 106	Fungi	M	19	7	nt	E	*fimG/fimH, escV*
	K 046	Fungi	M	26	4	O157	E	*fimG/fimH, escV, stx2*
	K 138	–	M	2	4	O127	A	*fimG/fimH, fyuA*
	K 096	–	F	3	1	O-rough	A	*fimG/fimH, fyuA*
	K 071	–	M	31	1	nt	A	*fimG/fimH, fyuA*
	K 044	Fungi	M	20	4	O125	A	*fimG/fimH, fyuA*
	K 064	Fungi	F	3	3	O126	A	*fimG/fimH, fyuA*
	K 028	Fungi	M	10	4	nt	F	*papC, fimG/fimH, fyuA*
	K 122	Fungi	M	17	3	O26	B1	*fimG/fimH, escV, fyuA*
	K 132	Fungi	F	15	2	O128	A	*fimG/fimH, astA, fyuA*
	K 027	*S. Enteritidis*, fungi	M	15	4	O126	A	*fimG/fimH, iutA*
	K 010	MSSA	F	3	4	O126	A	*fimG/fimH, iutA*
	K 029	Fungi	M	12	4	nt	E	*fimG/fimH, escV, iutA*
	K 015	–	M	8	3	nt	E	*fimG/fimH, astA, iutA*
	K 116	Fungi	M	12	1	O26	B1	*fimG/fimH, escV, fyuA, iutA*
	K 126	–	F	11	4	O26	B1	*fimG/fimH, escV, fyuA, iutA*
	K 008	–	M	8	4	O26	B1	*fimG/fimH, escV, fyuA, iutA*
	K 012	–	M	11	4	O26	B1	*fimG/fimH, escV, stx1, fyuA, iutA*
	K 142	MRSA	F	4	4	O119	F	*fimG/fimH, fyuA, iutA, iroN*
	K 011	Fungi	F	31	4	O125	A	*fimG/fimH, fyuA, iutA, iroN*
	K 074	*S. Enteritidis*	F	11	4	O25	F	*papC, fimG/fimH, fyuA, iutA, iroN*
	K 078	–	M	14	4	O25	F	*papC, fimG/fimH, fyuA, iutA, iroN*
	K 073	Fungi	F	32	4	nt	F	*fimG/fimH, fyuA, iutA, iroN, tsh*
	K 134	Fungi	F	30	4	O126	B1	*papC, fimG/fimH, fyuA, iutA, iroN, tsh*
	K 033	–	F	24	4	O124	A	*fimG/fimH, sat*
	K 014	–	M	4	2	O25	B2	*papC, sfaD/sfaE, cnf1, usp, hly1, fimG/fimH, fyuA, sat*
	K 051	Fungi	M	38	4	O86	A	*papC, hly1, fimG/fimH, pic, astA, iutA, sat*
	K 076	Fungi	M	17	4	O86	D	*fimG/fimH, fyuA, iutA, sat*
	K 127	Fungi	M	25	4	O86	A	*papC, hly1, fimG/fimH, pic, aggR, astA, fyuA, iutA, sat*
II	K 002	Fungi	M	30	4	O25	B2	*papC, fimG/fimH*
	K 120	Fungi	M	24	4	O114	B2	*fimG/fimH, escV*
	K 018	Fungi	F	5	4	O25	B2	*papC, fimG/fimH, iutA*
	K 021	Fungi	M	5	4	O25	B2	*papC, fimG/fimH, fyuA, iutA*
	K 060	Fungi	F	27	4	O25	B2	*papC, fimG/fimH, fyuA, iutA*
	K 025	–	F	33	4	O25	B2	*papC, cnf1, hly1, fimG/fimH, fyuA, iutA*
	K 034	–	M	8	4	O25	B2	*fimG/fimH, fyuA, iroN*
	K 035	–	M	8	4	O25	B2	*fimG/fimH, fyuA, iroN*
	K 005	MSSA	M	4	4	O25	B2	*papC, fimG/fimHfyuA, iroN*
	K 108	–	M	24	4	O25	B2	*fimG/fimH, astA, fyuA, iroN*
	K 043	Fungi	M	9	4	O25	B2	*papC, fyuA, iutA, iroN*
	K 036	Fungi	M	24	4	O25	B2	*papC, fimG/fimH, fyuA, iutA, iroN*
	K 141	*S. Enteritidis*	M	31	4	O25	B1	*papC, fimG/fimH, fyuA, iutA, iroN*
	K 023	Fungi	M	14	4	O25	B2	*papC, fimG/fimH, fyuA, iutA, iroN*
	K 137	MRSA	F	14	4	O25	B2	*papC, fimG/fimH, fyuA, iutA, iroN*
	K 115	Fungi	M	29	4	O25	B2	*papC, fimG/fimH, fyuA, iutA, iroN*
	K 001	Fungi	F	11	2	O25	B2	*papC, fimG/fimH, fyuA, iutA, iroN*
	K 017	–	F	9	4	O25	B2	*papC, fimG/fimH, fyuA, iutA, iroN*
	K 099	–	M	2	4	O25	B2	*papC, fimG/fimH, fyuA, iutA, iroN*
	K 117	MRSA	F	5	4	O25	B2	*papC, fimG/fimH, fyuA, iutA, iroN*
	K 124	*S. Enteritidis*, MSSA, fungi	F	25	4	O25	B2	*papC, fimG/fimH, fyuA, iutA, iroN*
	K 059	Fungi	M	7	4	O25	B2	*papC, fimG/fimH, fyuA, iutA, iroN*
	K 049	–	M	31	4	O25	B2	*papC, fimG/fimH, fyuA, iutA, iroN*
	K 052	–	M	17	4	O25	B2	*papC, fimG/fimH, fyuA, iutA, iroN*
	K 094	–	M	19	4	O25	B2	*papC, fimG/fimH, fyuA, iutA, iroN*
	K 009	–	F	1	4	O25	B2	*fimG/fimH, fyuA, iutA, tsh*
	K 082	Fungi	F	25	4	O25	B2	*fimG/fimH, fyuA, iutA, iroN, tsh*
	K 031	–	M	10	4	O25	B2	*fimG/fimH, fyuA, iutA, iroN, tsh*
	K 013	–	M	4	4	O25	B2	*fimG/fimH, fyuA, iutA, iroN, tsh*
	K 026	–	F	7	7	O25	B2	*fimG/fimH, fyuA, iutA, iroN, tsh*
	K 075	–	F	25	4	O25	B2	*fimG/fimH, fyuA, iutA, iroN, tsh*
	K 016	Fungi	M	8	7	nt	B2	*fimG/fimH, sat*
	K 030	Fungi	M	11	7	nt	B2	*fimG/fimH, sat*
	K 038	Fungi	F	28	4	nt	B2	*fimG/fimH, fyuA, iutA, sat*
	K 084	Fungi	M	31	4	O25	B2	*fimG/fimH, fyuA, iutA, sat*
	K 114	–	M	20	4	O25	B2	*fimG/fimH, fyuA, iutA, sat*
	K 067	–	M	31	4	O25	B2	*fimG/fimH, fyuA, iutA, sat*
	K 112	–	M	20	4	O25	B2	*fimG/fimH, fyuA, iutA, sat*
	K 055	Fungi	M	25	4	O25	B2	*fimG/fimH, fyuA, iutA, sat*
	K 039	MRSA	F	22	4	nt	B2	*fimG/fimH, fyuA, iutA, sat*
	K 129	Fungi	F	12	4	O25	B2	*fimG/fimH, fyuA, iutA, sat*
	K 110	–	F	10	4	O25	B2	*fimG/fimH, fyuA, iutA, sat*
	K 080	–	F	17	4	O25	B2	*fimG/fimH, fyuA, iutA, sat*
	K 085	MRSA, fungi	F	22	4	O25	B2	*fimG/fimH, fyuA, iutA, sat*
	K 061	–	F	23	4	nt	B2	*fimG/fimH, fyuA, iutA, sat*
	K 042	MSSA, fungi	M	18	6	O25	B2	*fimG/fimH, fyuA, iutA, sat*
	K 077	Fungi	M	20	6	O25	B2	*fimG/fimH, fyuA, iutA, sat*
	K 090	–	F	36	4	O25	F	*fimG/fimH, fyuA, iutA, sat*
	K 004	MSSA, fungi	M	27	4	O25	B2	*fimG/fimH, fyuA, iroN, sat*
	K 041	MSSA, fungi	F	21	4	O25	B2	*papC, fimG/fimH, fyuA, iutA, iroN, sat*
III	K 102	–	F	31	4	nt	D	*fimG/fimH*
	K 093	Fungi	F	33	4	O44	D	*fimG/fimH, fyuA*
	K 140	–	M	6	4	O142	B1	*fimG/fimH, astA, iroN*
	K 062	–	M	7	2	O25	B2	*papC, sfaD/sfaE, cnf1, usp, hly1, fimG/fimH, fyuA, iutA, iroN*
	K 066	–	F	13	2	O44	D	*fimG/fimH, sat*
	K 020	Fungi	M	23	4	nt	D	*fimG/fimH, sat*
	K 022	Fungi	F	14	4	nt	D	*fimG/fimH, sat*
	K 003	Fungi	M	18	4	O86	D	*papC, fimG/fimH, astA, sat*
	K 040	Fungi	M	19	4	nt	D	*fimG/fimH, iutA, sat*
	K 089	Fungi	F	8	2	nt	D	*fimG/fimH, iutA, sat*
	K 104	–	F	31	4	O44	D	*fimG/fimH, iutA, sat*
	K 111	Fungi	F	22	2	nt	D	*fimG/fimH, iutA, sat*
	K 097	Fungi	F	17	2	nt	D	*fimG/fimH, iutA, sat*
	K 113	–	M	33	3	nt	D	*fimG/fimH, iutA, sat*
	K 065	–	F	13	4	nt	D	*fimG/fimH, iutA, sat*
	K 083	Fungi	F	16	2	nt	D	*fimG/fimH, iutA, sat*
	K 079	fungi	F	23	4	nt	D	*fimG/fimH, iutA, sat*
	K 095	–	F	31	4	nt	D	*fimG/fimH, iutA, sat*
	K 063	Fungi	F	24	3	nt	D	*fimG/fimH, iutA, sat*
	K 081	–	M	24	4	nt	D	*fimG/fimH, iutA, sat*
	K 087	Fungi	F	21	3	nt	D	*fimG/fimH, iutA, sat*
	K 086	MRSA, *S. Enteritidis*, fungi	F	36	4	O44	D	*fimG/fimH, iutA, sat*
	K 024	–	F	20	4	nt	D	*fimG/fimH, fyuA, iutA, sat*
	K 072	*Y. enterocolitica*., fungi	F	7	4	O44	D	*papC, fimG/fimH, fyuA, iutA, sat*
	K 091	–	F	18	4	nt	D	*papC, fimG/fimH, fyuA, iutA, sat*
	K 019	Fungi	M	20	5	nt	D	*fimG/fimH, fyuA, iutA, iroN, sat*
	K 006	–	M	15	4	nt	D	*fimG/fimH, fyuA, iutA, iroN, sat*

^a^M/F—male/female

^b^Place of isolation—different numbers refer to different regions; *1* Aleksandrow Lodzki, *2* Dlutow, *3* Leczyca, *4* Lodz, *5* Piotrkow Trybulanski, *6* Radomsko, *7* Tuszyn

^c^ *nt* non-typable

(−) none of the studied virulence factors identified

### Bacterial growth and genomic DNA isolation

All of the *E. coli* strains were grown in a liquid LB broth at 37 °C with agitation (120 RPM) for 24 h. Genomic DNA isolation and purification was performed with the use of a GenElute Bacterial Genomic DNA Kit (Sigma-Aldrich, St. Louis, MO). The quantity and purity of each genomic DNA sample was determined spectrophotometrically at 260 nm (BioPhotometer, Eppendorf, Germany). The DNA samples were diluted to 20 ng/µl and then used.

### Phylogenetic structure

Assignment of a phylogenetic group was carried out according to Clermont’s *E. coli* phylo-typing improved method [8]. Sequences of primers, their concentrations and phylo-group detection schemes were conducted strictly as described in the new quadruplex phylo-group assignment method [8].

### Virulence factors

The presence of uropathogenic virulence factors was determined using sequences of primers previously published [10]. Detection of pathovar target genes (intestinal virulence factors) was performed according to the Müller et al. protocol [5].

Two separate PCRs were performed in order to detect the presence of an additional five virulence genes. The first multiplex was composed for determination of *iroN*, *fyuA* and *iutA* presence. Sequences of primers for *iroN* detection as published by Bonacorsi et al. [11], and sequences of primers for detection of *fyuA* and *iutA* as published by Johnson and Stell [12], were employed. The second one was composed for determination of *sat*, as described by Restieri et al. [13], and for *tsh* presence, as described by Moulin-Schouleur et al. [14].

All of the amplifications were performed according to manufacturers’ guidelines for The Platinum^®^ Multiplex PCR Master Mix (Thermo Fisher Scientific, Foster City, CA) in a T-3000 thermocycler (Biometra, Goettingen, Germany). Amplification products were separated on 1.6 % agarose gels in 1× TAE buffer at room temperature until the dye (bromophenol blue) migrated 6 cm from the beginning of the gel (2.4 V/cm), ethidium bromide stained, photographed and analyzed.

### TRS-PCR and fingerprint analysis

The conditions for amplification of the TRS profiles, including primer sequences, electrophoresis, reproducibility analysis, determination of diversity indices and bioinformatic analyses were performed as described elsewhere [10, 15–18]. One exception was DNA concentration—for each amplification, 20 ng of genomic DNA was used. For each strain, two fingerprint types based on the presence of CGG or GTG motif were generated. The PCRs were performed in a T3000 Biometra thermal cycler. Amplification products were separated on 1.6 % agarose gels in 1× TAE buffer, ethidium bromide stained and photographed. Subsequently, gels were optimized according to recommendations provided by BioNumerics software (Applied Maths, Belgium) and normalized with regard to a 100 bp Plus DNA size marker (Fermentas, Thermo Scientific Waltham, MA, USA). The composite (mean) fingerprint similarity analysis based on CGG-PCR and GTG-PCR fingerprints using Pearson correlation (optimization 1 %, position tolerance 1 %) and grouping according to the UPGMA algorithm was performed.

## Results

### Phylogenetic structure and virulence profiling of the *E. coli* collection

The distribution of phylogenetic groups among *E. coli* strains was as follows: 20 strains were represented by phylogroup A, 16 by B1, 50 by B2, 26 by D, 5 by E and 6 strains were represented phylogroup F. Phylogroup C was not detected in the collection. All this information is gathered in Table 1.

A virulence profile was defined for each of the 123 *E. coli* strains (Table 1). Among this group of strains, 39 unique virulence profiles were identified, which makes this collection very heterogeneous. Three profiles—*fimG/fimH*, *fyuA*, *iutA*, *sat* (17 strains), *papC*, *fimG/fimH*, *fyuA*, *iutA*, *iroN* (16 strains) and *fimG/fimH*, *iutA*, *sat* (14 strains)—were predominant (Table 1). What is interesting is that 11 strains had a single-gene virulence profile—*fimG/fimH*—and 2 strains had none of the analyzed virulence genes (Table 1).

### TRS-PCR and fingerprint analysis

The (CGG)_4_- and (GTG)_4_-based PCR tests were conducted on a collection of 123 *E. coli* strains. Both tests generated fingerprints for each strain. Reproducibility analyses for these tests were calculated and found to be at a very similar level. When used separately, it was 96.2 % for CGG-PCR and 95.0 % for GTG-PCR. For the composite analysis, it exhibited 95.0 %. Two separate analyses of fingerprint similarity were made for both TRS-PCR tests and diversity indices (DI) according to the Hunter and Gaston algorithm [19] that had been calculated for them. The DI value for CGG-PCR and GTG-PCR were 0.987 and 0.951, respectively.

Based on that, a composite analysis of CGG-PCR and GTG-PCR fingerprint similarity was conducted. For this analysis, the diversity index (DI) had been calculated. Its value was high—0.991—which confirms the utility of the applied test for diversification of those *E. coli* strains. The calculated parameters allowed for distinguishing 87 clusters, which means 87 unique fingerprints for 123 isolates.

**Fig. 1:**
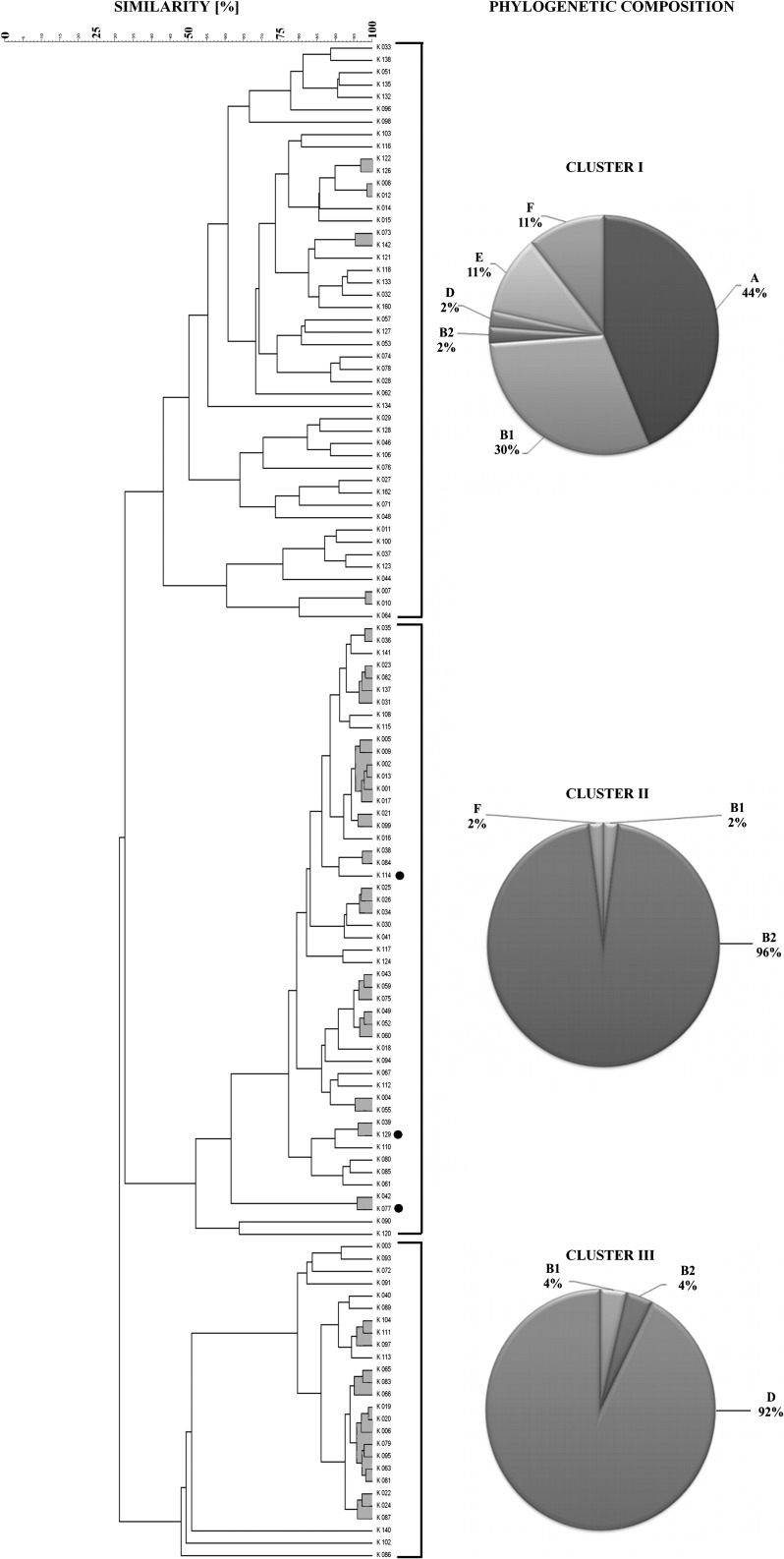
The composite CGG-TRS and GTG-TRS fingerprints similarity comparison of 123 *E. coli* strains isolated from children with diarrhea and phylogenetic composition within clusters. *Black dots* indicate an example of three strains with identical virulence factors, the same phylogroup and O-antigen. *Grey zones*—strains with identical TRS-PCR profiles. The similarities between fingerprints were calculated using Pearson correlation (optimization 1.00 %, position tolerance 1.00 %) and fingerprints were grouped by use of the UPGMA algorithm

**Fig. 2:**
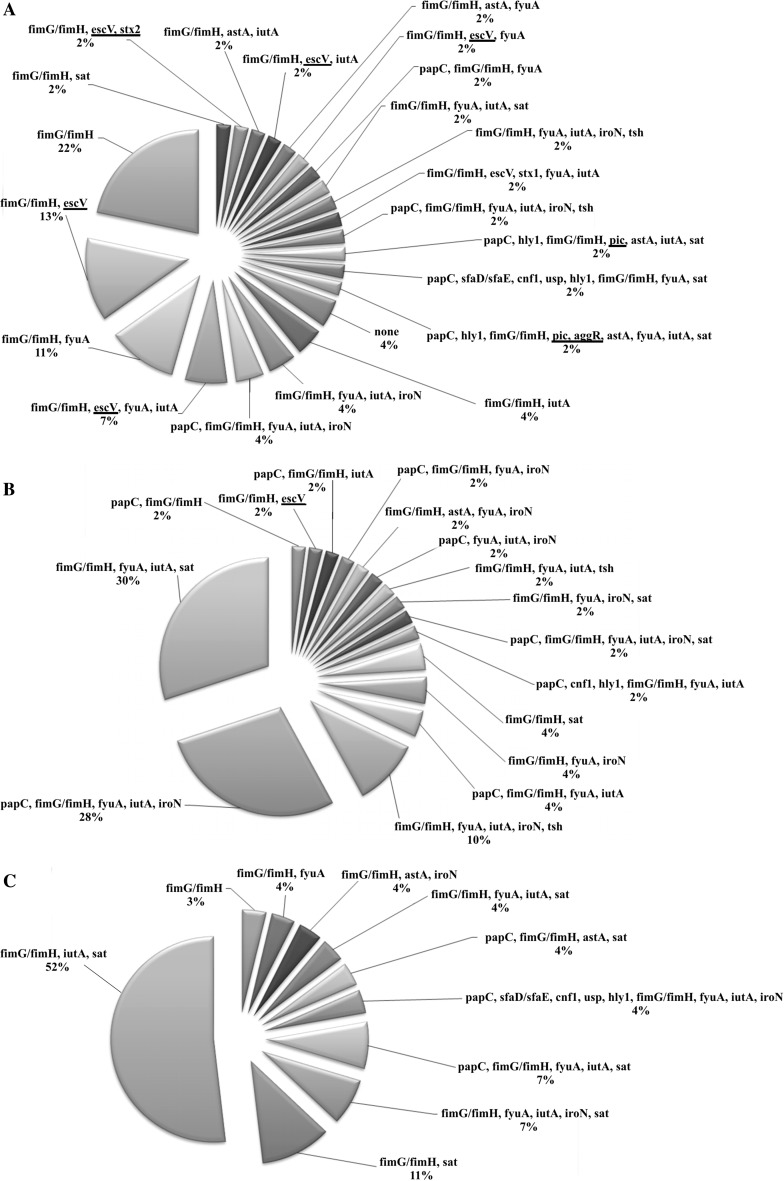
Virulence profiles within clusters (**A** - cluster I, **B** - cluster II, **C** - cluster III) of *E. coli* strains

Taking this composite analysis into consideration, one may notice the strains grouped into three separate clusters (Fig. 1). These clusters were different with regard to phylogenetic structure and virulence profiles. Inter-cluster similarities were 43.36, 51.58 and 47.47 %, respectively. Among the first cluster, 44 % of the strains were represented by phylogroup A and 30 % by B1. Also, there were strains belonging to phylogroup E (11 %), F (11 %), B2 (2 %) and D (2 %) (Table 1; Fig. 1). What is more, there were 22 different virulence profiles for 46 strains in this cluster (Fig. 2).

The phylogenetic structure in the second cluster strains was as follows: 96 % of the strains belonged to phylogroup B2, one strain to B1 and one to phylogroup F (Table 1; Fig. 1). In the second cluster, there were 50 strains, revealing 16 different virulence profiles (Fig. 2.).

Analysis of the third cluster revealed that most of the strains belonged to phylogenetic group D (92 %). There were strains representing phylogroup B1 and B2, 4 % each (Table 1; Fig. 1). Twenty-seven strains from this cluster had 9 distinguishing virulence profiles (Fig. 2.).

## Discussion

Infections due to enteropathogens still pose a serious threat in many regions of the world. Children from high- and low-income countries are at the center of attention of many authorities from the viewpoint of diarrheal diseases [2, 3, 9]. It is known that in the case of children, enteric infections are not always caused by one pathogen or that the detected pathogen is the cause of the illness [9]. In our study concomitant infections were also observed (Table 1). To accomplish guidelines resulting from WHO’s GAPPD project [3] and taking into consideration many statistics associated with children’s morbidity and mortality [4], there are many issues that may be done in the field of *E. coli* infections in children.

Some researchers underline that studies on DECs are very needed, not only to widen the general knowledge about these strains but also to follow changes in new emerging pathotypes, which will be useful in making epidemic predictions [1, 2, 9]. From our point of view, improving and implementing more accurate and easy methods useful in epidemiology of pathogenic *E.* *coli* strains are among such activities. We showed earlier that TRS-PCR was an effective tool for differentiation of UPEC strains [10]. In this paper, composite analysis based on CGG-PCR and GTG-PCR fingerprints was conducted on a collection of *E. coli* strains isolated from children with diarrhea (Fig. 1). From the group of 10 CG-rich TRS primers, we chose those with CGG and GTG motifs. CGG-PCR was effectively used for discrimination of the UPEC strains [10], so we assumed that it could also be useful in the case of intestinal *E. coli*. The GTG-PCR was chosen as it was successfully employed in our laboratory for inter-serovar discrimination of *Salmonella* strains [15]. What is more, (GTG)_5_-PCR tests were used for genotyping *Klebsiella* strains [20] and identification of *Streptococcus mutans*, *Staphylococcus* spp. and *Enterococcus* spp [21–23]. In this work, we decided on composite analysis of these two tests because, in general, composite analysis of two methods yields better results. This fact was confirmed by DI values—the highest value was obtained for the composite analysis demonstrating 88 unique TRS profiles (Fig. 1).

Taking the structure of the dendrogram into consideration, it can be noticed that fingerprints obtained for this collection of *E. coli* might be used for strain diversification. This was verified by the DI value of 0.991 and high reproducibility (95.0 %). Noteworthy, CGG-PCR and GTG-PCR profiles for these strains may enable phylogenetic investigations. Distribution of the phylogenetic groups represented by tested isolates within the dendrogram differed between three distinct clusters. Most of the strains in the first cluster represented phylogroup A and B1 (74 %) (Fig. 1). Strains belonging to phylogroup B2 were predominant in the second cluster (96 %), and those belonging to phylogroup D prevailed in the third cluster (92 %) (Fig. 1). Strains of phylogroups B2 and D are more typical of those that cause extraintestinal infections, while intestinal pathogens more often belong to phylogroup D than B2, B1 or A [24]. Commensal strains of *E. coli* generally represent phylogroups A and B1—potentially non-harmful intestinal isolates [24]. As our study shows, virulence factors typical for IPEC were rarely detected in our collection, but other virulence factors were found (Table 1). When analyzing the dendrogram presented in Fig. 1, one may notice that strains encoding virulence factors typical for IPEC were mostly grouped in the first cluster (15 of the 16 strains). However, this cluster also includes strains that do not have any of these VFs. Because strains in this cluster represented phylogenetic groups typical for intestinal isolates, one may suppose that this is a mixed group with potential pathogens and commensal strains. Commensal *E. coli* strains seldom are causes of diseases, but the exceptions are debilitated or immunocompromised hosts or sensitive individuals, for example, after previous antibiotic treatment or those with some bowel diseases [6, 7, 9]. Many of the tested isolates were derived from children with some additional microbiological infections (Table 1), but we had no information about other diseases or viral infections. Thus, since the morbidity also depends on the condition of the host, and bearing in mind all that is mentioned above, it is obvious that children are a very specific target for intestinal infections due to *E.* *coli* and that even a potentially non-harmful strain may, in convenient conditions, become a nagging pathogen and cause dangerous diarrhea.

In this paper, we propose a complex approach to genotyping of *E. coli* isolated from children with diarrhea, in which the presence of many virulence factors and TRS-PCR profiles were determined. It should be underlined that virulence factors are not restricted to one pathovar and can occur in other pathotypes, which influence the virulence potential of the strain [5]. This indicates that epidemiological assays should not be based on virulence profiling only. Our complex approach allowed for better diversification of strains encoded with the same virulence profiles and, in some cases, which belonged to the same phylogenetic group yet had different TRS-PCR fingerprints (marked in Fig. 1). However, the strains with identical TRS-PCR profiles and different VF set such as: K001, K002, K009, K013 and K017 or K035 and K036, should be additionally sub-typed. Analyzes employing other TRS primers demonstrated, that some of these strains could be separated by CAC-PCR (data not shown). It cannot be excluded that TRS-PCR profiling might not be sufficient for a full differentiation, therefore should be combined with virulence gene detection, serogrouping or phylogenetic affiliation [25].

For molecular diagnostics and DNA-based strain typing such methods as PFGE, MLVA or MLST are routinely used. Without comparative analyzes, it is hard to asses if TRS-PCR profiling has superior power to the these methods. Accepting that TRS-PCR has comparable resolution, it is still inexpensive, rapid and easy applicable among routinely used approaches. The obtained results support and are consistent with the need of public health surveillance for searching new and fast assays as far as children’s health is concerned. To sum up, TRS-PCR profiling is an effective tool for genotyping *E. coli* strains isolated from children with diarrhea.
